# Co-toxicity of Endotoxin and Indoxyl Sulfate, Gut-Derived Bacterial Metabolites, to Vascular Endothelial Cells in Coronary Arterial Disease Accompanied by Gut Dysbiosis

**DOI:** 10.3390/nu14030424

**Published:** 2022-01-18

**Authors:** Marcin Choroszy, Beata Sobieszczańska, Kamil Litwinowicz, Łukasz Łaczmański, Mateusz Chmielarz, Urszula Walczuk, Tomasz Roleder, Jadwiga Radziejewska, Magdalena Wawrzyńska

**Affiliations:** 1 Department of Microbiology, Wrocław Medical University, Chalubinskiego 4 Street, 51-657 Wroclaw, Poland; marcin.choroszy@student.umw.edu.pl (M.C.); mateusz.chmielarz@student.umw.edu.pl (M.C.); urszula.walczuk@umw.edu.pl (U.W.); 2 Department of Medical Biochemistry, Wroclaw Medical University, Chalubińskiego 10 Street, 50-368 Wroclaw, Poland; kamil.litwinowicz@student.umw.edu.pl; 3 Laboratory of Genomics & Bioinformatics, Institute of Immunology and Experimental Therapy, Polish Academy of Sciences, Weigla 12 Street, 53-114 Wroclaw, Poland; lukasz.laczmanski@hirszfeld.pl; 4 Research and Development Centre, Regional Specialist Hospital, Kamienskiego 73a Street, 51-124 Wroclaw, Poland; tomasz.roleder@wssk.wroc.pl; 5 Klodzko County Hospital, 57-300 Kłodzko, Poland; radziejewska.jadwiga@zoz.klodzko.pl; 6 Department of Preclinical Studies, Faculty of Health Sciences, Wrocław Medical University, 50-367 Wrocław, Poland; magdalena.wawrzynska@umw.edu.pl

**Keywords:** coronary artery disease, gut microbiome, dysbiosis, obesity, *Bacteroidetes*, LPS, indoxyl sulfate

## Abstract

Gut dysbiosis, alongside a high-fat diet and cigarette smoking, is considered one of the factors promoting coronary arterial disease (CAD) development. The present study aimed to research whether gut dysbiosis can increase bacterial metabolites concentration in the blood of CAD patients and what impact these metabolites can exert on endothelial cells. The gut microbiomes of 15 age-matched CAD patients and healthy controls were analyzed by 16S rRNA sequencing analysis. The in vitro impact of LPS and indoxyl sulfate at concentrations present in patients’ sera on endothelial cells was investigated. 16S rRNA sequencing analysis revealed gut dysbiosis in CAD patients, further confirmed by elevated LPS and indoxyl sulfate levels in patients’ sera. CAD was associated with depletion of *Bacteroidetes* and *Alistipes*. LPS and indoxyl sulfate demonstrated co-toxicity to endothelial cells inducing reactive oxygen species, E-selectin, and monocyte chemoattractant protein-1 (MCP-1) production. Moreover, both of these metabolites promoted thrombogenicity of endothelial cells confirmed by monocyte adherence. The co-toxicity of LPS and indoxyl sulfate was associated with harmful effects on endothelial cells, strongly suggesting that gut dysbiosis-associated increased intestinal permeability can initiate or promote endothelial inflammation and atherosclerosis progression.

## 1. Introduction

Coronary artery disease (CAD) includes stable and unstable angina, myocardial infarction, and sudden cardiac death, the primary cause of morbidity and mortality worldwide [1]. The mechanisms and risk factors involved in the pathogenesis of CAD have been well-documented over recent decades. However, early inducers triggering the cascade of inflammation and atherosclerotic plaques formation are only putative.

Gut dysbiosis related to the change in diversity and abundance of resident intestinal microbiota plays a vital role in cardiovascular diseases [2,3]. Gut dysbiosis induces chronic inflammation resulting in increased intestinal epithelial permeability and leakage of bacterial metabolites into the bloodstream [4]. Some of these metabolites can activate endothelial cells and promote atherosclerosis. Moreover, bacterial metabolites may interact with each other increasing their mutual cytotoxicity to the endothelial cells directly or indirectly via the effect on monocyte/macrophage.

To date, few bacterial metabolites that exert deleterious effects on vascular endothelium have been recognized. One of those is indole, a tryptophan derivative produced by gut microbiota, oxidized to indoxyl sulfate (IS) in the liver and then removed from the body throughout kidneys filtration. The more indole is produced in the intestine, the more is oxidized in the liver and released into circulation. Elevated indoxyl level induces oxidative stress, pro-inflammatory response, and enhanced expression of adhesion molecules in endothelial cells. The toxic effect of indoxyl on endothelial cells contributes to chronic kidney disease (CKD) and is involved in cardiovascular diseases pathogenesis in CKD patients [5]. However, the impact of indoxyl on CAD pathogenesis in patients without renal impairment is still lacking.

Another well-known bacterial metabolite with deleterious effects on the endothelial cells is endotoxin (LPS), originating from the outer membrane of Gram-negative bacteria. A high-fat diet increases intestinal permeability resulting in metabolic endotoxemia. Continuous translocation of low doses of LPS from intestines into circulation eventually leads to endotoxin tolerance and the development of chronic hypoinflammation identified as a casual of atherosclerosis and many other diseases [6]. LPS influences the endothelium indirectly via pro-inflammatory cytokines or affects endothelial cells directly. In vascular endothelium, LPS upregulates nitric oxide synthase (iNOS), increases endothelium permeability, and enhances the expression of leukocytes’ adhesion molecules.

Significant risk factors for the development of CAD are lipoproteins and the high-fat diet as their source. Low-density lipoproteins (LDL) entering arterial intima from the blood are considered the primary driver of atherogenesis [7]. Diet is also an essential factor regulating the composition and metabolic activity of the intestinal microbiota. Hence, an unhealthy diet results in gut dysbiosis and leakage of bacterial metabolites into the circulation [8]. Furthermore, LPS induces the conversion of the endothelial surface from anti-coagulant to pro-coagulant and affects multiple signaling pathways via the TLR4 receptor [9]. Plasma lipoproteins such as HDL, LDL, and VLDL can sequester LPS from circulation to limit its harmful impact on the host cells [10]. LPS sequestration by plasma lipids, however, may become a pathway of its delivery into monocytes/macrophages infiltrating vascular endothelium activated by cytokines released by monocytes in response to bacterial metabolites. Moreover, at the site of clot formation, LPS alone or combined with indoxyl via ROS formation in ECs can promote lipids oxidation and the formation of foamy macrophages.

The present study focused on the association of gut dysbiosis and an in vitro analysis of the direct and indirect effects of indoxyl and LPS at the concentrations present in the sera of dysbiotic CAD patients on vascular endothelial cells. The study results demonstrated gut dysbiosis in CAD patients and elevated levels of bacterial metabolites, i.e., LPS and indoxyl sulfate in their sera. In vitro co-toxicity of LPS and indoxyl sulfate in meager concentrations was associated with deleterious effects on endothelial cells, strongly suggesting that overweight-associated increased intestinal permeability can initiate or promote endothelial inflammation.

## 2. Materials and Methods

### 2.1. Study Participants

Fifteen CAD patients and 15 healthy individuals were enrolled in the study. CAD was confirmed by coronary angiography, and patients with ≥50% stenosis in single or multiple vessels were qualified for the study. Study participants were recruited at Regional Specialist Hospital between March and September 2020. Biochemical parameters were assessed employing standard techniques at the Specialist Hospital’s Laboratory (Table 1). The exclusion criteria included renal disease defined as an abnormal creatinine serum level (>2 mg/dL), malignancy, ongoing infectious disease, hepatic disease, and use of antibiotics within four weeks before sample collection. Unfortunately, in the study, there are sex ratio differences between the CAD and control groups. CAD affects men more often than women; hence the patient group included mainly men. However, obtaining stool samples from healthy older adults for the study turned out unexpectable difficult. For unknown reasons, perhaps embarrassment, men usually refused to provide stool samples, so the control group included mainly women. All study participants gave their written consents; the study followed guidelines of the Helsinki Declaration and was approved by Wroclaw Medical University’s Ethics Committee (authorization number KN-209/2020).

### 2.2. Microbiome Analysis

Stool samples from all participants, collected and transported to the laboratory in an ice bag, were stored at −80 °C until processed. DNA was extracted from stool specimens using the QIAamp DNA Stool Mini Kit. The hypervariable V3-V4 regions of the 16S ribosomal RNA (rRNA) gene were amplified. Quantitative real-time polymerase chain reaction amplification was performed in triplicate. The products were purified, and then pyrosequencing was performed using the MiSeq system (Illumina, San Diego, CA, USA). The entire workflow for microbiome analysis was run in Snakemake [11], cutadapt [12], USEARCH3 algorithm [13], phyloseq object [14], decipher idtaxa [15], with SILVA as reference database [16]. 

### 2.3. Endotoxin, Indoxyl Sulfate, Measurements in Patients Sera

LPS level was determined using PierceTM LAL chromogenic assay (ThermoFisher Science, Basel, Switzerland), whereas IS was measured in plasma samples with Human Indoxyl Sulphate ELISA Kit (MyBioSource, Bergkällavägen, Sweden). Both assays were performed according to the manufacturer’s instructions.

### 2.4. Cell Cultures and Conditioned Medium Preparation

Endothelial cells (primary human umbilical vein endothelial cell line HUVEC C-015-10C was obtained from ThermoFisher Scientific) were routinely cultured in an EBM-2 bullet kit medium (Lonza, Cologne, Germany). For experiments, HUVECs were trypsinized and seeded into cell culture plates at the density of 4 × 10^5^ cells/mL in M199 medium (ThermoFisher Science, Basel, Switzerland ) supplemented with 10% fetal bovine serum (FBS) and cultured overnight. The cells between passages 2 and 4 were used in the study. The human monocytic THP-1 cell line (EP-CL-0233) from Elabscience was cultured in RPMI-1640 medium (ThermoFisher Science, Basel, Switzerland) with 10% FBS and 1% antibiotics solution (penicillin, streptomycin). THP-1 cells were differentiated into monocyte-derived macrophages (MDM) with 50 ng/mL PMA (phorbol 12-myristate 13-acetate; Merc Life Science, Darmstadt, Germany) for 48 h. The obtained monocyte-derived macrophages (MDM) were stimulated for 18 h with LPS (from *Salmonella typhimurium*) and indoxyl sulfate (both from Merc Life Science, Darmstadt, Germany) at concentrations corresponding to a mean concentration detected in patients sera and ten times higher concentrations. Thus, LPS was used at concentrations 3 ng/mL or 30 ng/mL and indoxyl at 13 µM and 130 µM. The mixtures of both metabolites at concentrations LPS 3 ng/mL + indoxyl 13 µM, LPS 30 ng/mL + indoxyl 130 µM were used to assess the co-toxicity of these both metabolites. Every time, before cell treatment, LPS and IS after dilution in a cell culture medium were preincubated for 1 h at 37 °C to interact with serum proteins, i.e., albumin for IS and LPS-binding protein (LBP) for LPS. The negative control included MDM in RPMI-1640 medium without LPS and indoxyl. After MDM stimulation with LPS and/or IS, the culture medium was drawn and centrifuged for 10 min at 1500× *g* rpm and filter-sterilized (0.22 µm). The resultant supernatants (called conditioned media; CM) were frozen at −70 °C for further study. The CM from macrophages apart from LPS and indoxyl contains cellular metabolites and cytokines in concentrations toxic to cultured cells. Hence, CM was diluted with a cell culture medium to a concentration of 25% (hereafter referred to as CM25).

### 2.5. Cells Viability and ROS Measurement

HUVECs and MDM viability was assessed with 1 mg/mL MTT (3-(4,5-dimethylthiazol-2-yl)-2,5-diphenyl tetrazolium bromide; Merc Life Science, Darmstadt, Germany). The cells were treated with LPS and IS at appropriate concentrations for 18 h and followed with incubation with MTT for 2 h at 37 °C in an atmosphere with 5% CO_2_. The MTT solution was removed, and 100 µL DMSO was added to wells to dissolve violet formazan crystals. The reaction was read at 570 nm in a spectrophotometer UV/VIS 340. Cell morphology was assessed under an inverted microscope and after staining with phalloidin-FITC and DAPI to visualize the cell actin filaments and nuclei, respectively. The 2′, 7′-dichlorodihydrofluorescein diacetate (H2DCF-DA; Merc Life Science, Darmstadt, Germany) was used to assess ROS production in HUVECs treated with LPS and IS at appropriate concentrations for 5 h. HUVECs treated with 50 µM H_2_O_2_, and untreated cells served as a positive control and negative control, respectively. After treatment, cells were washed three times with Hank’s Balanced solution (HBSS), and 10 µM H2DCF-DA in HBSS was added to cells for 30 min. The fluorescent product was quantified with a spectrophotometer Tecan Infinite M200 plate reader at 488/525 nm.

### 2.6. Endothelial Cells Thrombogenicity Assessment

The effect of LPS and indoxyl on HUVECs thrombogenicity was investigated by monocyte adherence, E-selectin, and MCP-1 investigation. HUVECs were pre-treated for 5 h with LPS and indoxyl at appropriate concentrations in M199 medium supplemented with 10% FBS and CM25 diluted in M199 medium. THP-1 cells at the density 1 × 10^6^ cell/mL were stained with 10 µg/mL calcein-AM for 1 h and added to stimulated HUVEC cells for 4 h. Following three washes in HBSS, the number of THP-1 cells adhering to HUVECs was quantified under a fluorescence microscope (from six different fields of view) and expressed as the percentage of adhering monocytes relative to negative control, i.e., unstimulated HUVEC cells, considered 100%.

E-selectin level in HUVEC cells stimulated with LPS and indoxyl, and CM25 was assessed according to the protocol described by Grabner et al. [17]. Briefly, confluent HUVECs layers in a 6-well plate stimulated with CM25 or LPS and IS for 18 h was washed twice with PBS and fixed with cold 0.5% formalin in PBS for 2.5 min. Then, cells were washed with HBSS-BSA (0.5% *w*/*v*) before adding FITC-conjugated mouse monoclonal anti-human E-selectin antibody (R&D Systems, Abingdon, United Kingdom) at concentration 10 µg per 1 × 10^6^ cells in HBSS with 0.02% saponin (*w*/*v*). The reaction was run for 45 min at 4 °C in darkness, followed by two washes in HBSS-saponin and detachment with 0.25% trypsin. Detached cells were collected and centrifuged at 1000× *g* rpm at 4 °C for 10 min, washed twice with cold PBS, and finally diluted in PBS. A 100 µL sample was added to a 96-well black-walled plate in triplicate and read using a Tecan Infinite M200 plate reader at 488/535 nm. HUVECs incubated at the same conditions with an isotype mouse FITC-conjugated antibody (Merc Life Science, Darmstadt, Germany) served as a negative control.

MCP-1 protein was assessed in the culture media from HUVECs stimulated with CM25 and LPS and IS antigens for 5 h using the MCP-1 Human ELISA kit (ThermoFisher Science, Basel, Switzerland) performed according to the manufacturer’s instruction. The level of MCP-1 was calculated from a standard curve.

### 2.7. Statistical Analysis

For alpha diversity analyses, reads were normalized to 5000 reads by subsampling without replacements. Alpha diversity was assessed using Chao1 and Fisher’s alpha (richness), Bulla and Simpson indices (evenness), and dominance index. In addition, beta-diversity was assessed using principal coordinate analysis (PCoA) weighted UniFrac, unweighted UniFrac, and Bray–Curtis distances. Statistical significance was tested with PERMANOVA using 9999 permutations. All in vitro study data are presented as the mean ± SEM from at least three independent experiments performed in triplicate unless otherwise indicated in the figure legend. Statistical significance of differences between means was determined by analysis of variance (one-way ANOVA with post hoc Tukey’s honestly significant difference) and Wilcoxon signed-ranks test with *p ≤* 0.05 considered statistically significant. The differences in enterotypes distribution in study groups were analysed using the chi-square test with *p* ≤ 0.05 considered statistically significant.

## 3. Results

Bacterial metabolites leaking from the gut into the bloodstream can affect the vascular endothelium directly or indirectly by factors released from white blood cells upon contact with bacterial antigens. To better understand the role of gut dysbiosis in the development of atherosclerosis, the study aimed to evaluate, first, the gut microbiome in CAD patients and healthy controls, second, the levels of LPS and IS in the sera of CAD patients and their direct and indirect effects on the vascular endothelium.

**Figure 1:**
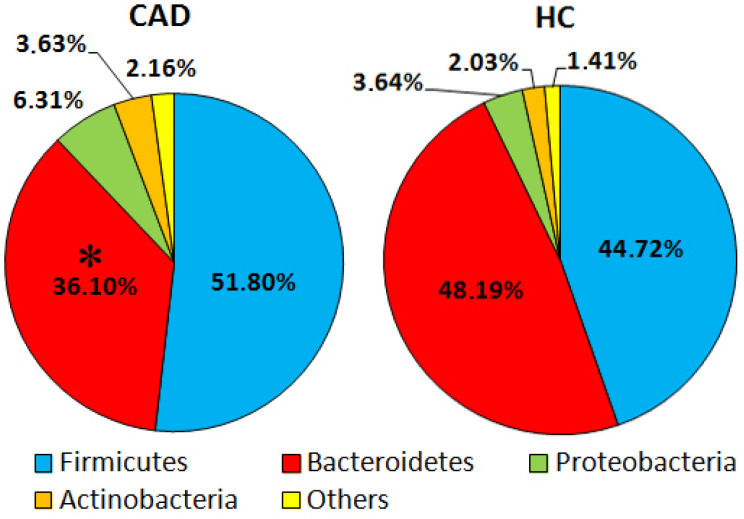
Community abundance (%) in gut microbiome of CAD patients and healthy controls (HC) at the phylum level. The asterisk indicates a statistically significant difference (*p* < 0.05) between CAD and HC groups.

**Figure 2:**
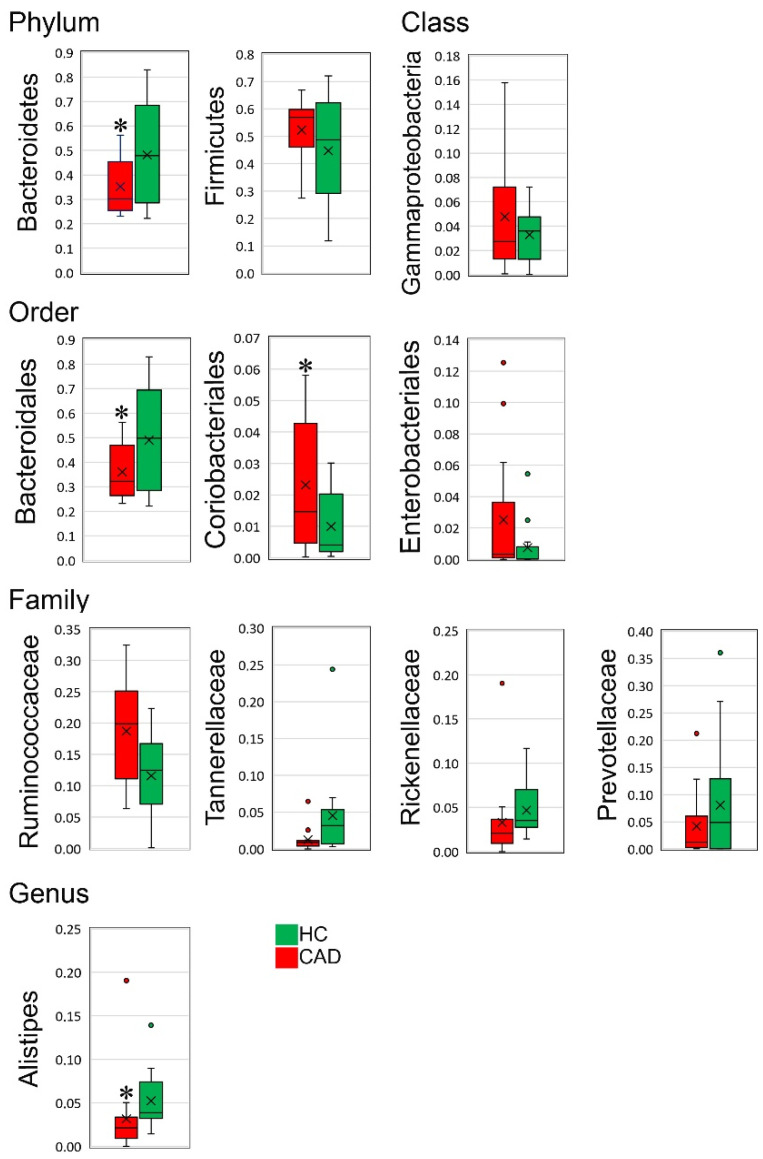
The most notable differences at the phylum, class, order, family and genus levels in the gut microbiome of CAD patients compared to healthy controls. The asterisks above bars indicate statistically significant differences (*p* < 0.05) between CAD and HC groups.

There are five core bacterial phyla within the gut microbiome. The most numerous *Firmicutes* make up 65%, followed by *Bacteroidota* accounting 30%, *Proteobacteria* and *Verrucomicrobiota* constituting 2%, *Actinobacteria comprising* 1%, and other phyla contributing <1% [18]. In the study, the 16S rRNA analysis revealed noteworthy differences in gut microbiome composition at the phylum, class, order, family, and genus levels in CAD patients compared to a group of healthy subjects (Figure 1 and Figure 2). At the phylum level, the most striking difference was *Bacteroidetes* depletion in CAD patients compared to HC (*p* = 0.01). In addition, upward trends in *Firmicutes*, *Proteobacteria*, and *Actinobacteria* were observed in the CAD group compared to HC, although they were statistically insignificant (Figure 1). *Gammaproteobacteria* also trended upward in the CAD group at the class level compared to the HC group. Order-level microbiome analysis confirmed a significant reduction in *Bacteroidales* (*p* = 0.03), a significant increase in *Coriobacteriales* (*p* = 0.04), and an upward trend in *Enterobacteriales* in CAD patients to healthy subjects. The analysis at the family level indicated *Rickenellaceae*, *Tannerelaceae*, *Prevotellaceae* depletion, and *Ruminococcaceae* over-representation in CAD patients compared to the HC group. However, the differences were statistically insignificant, most probably due to the small size of the study. At the genus level, a significant depletion of *Alistipes* of the *Rickenellaceae* family was recorded in CAD compared to HC (*p* = 0.01) (Figure 2). 

**Figure 3:**
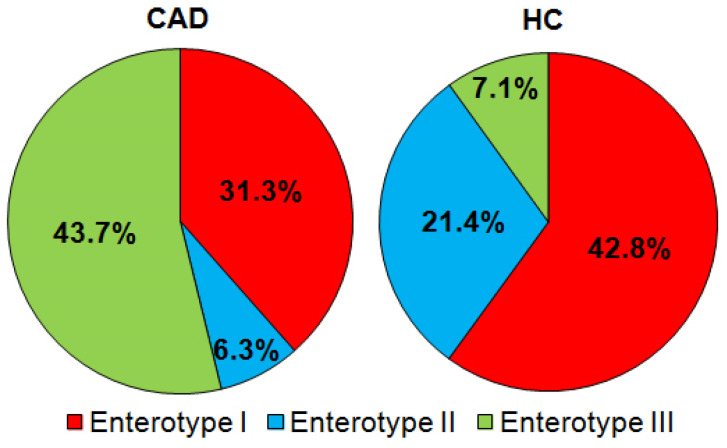
Enterotypes (%) distribution in CAD and HC groups. Enterotype I—Bacteroides prevalence; enterotype II—prevotella prevalence, and enterotype III Ruminococcus prevalence.

Three enterotypes have been distinguished in the human gut microbiome based on the abundance of key bacterial genera, i.e., high in *Bacteroides* enterotype I, high in *Prevotella* enterotype II, and enterotype III characterized by a high level of *Ruminococcaceae* [19,20]. In the study, enterotype II was under-represented in CAD patients (*p* < 0.0001) compared to HC. In contrast, enterotype III was over-represented in CAD (*p* < 0.0001), confirming the appreciable differences in the gut microbiome between CAD patients and healthy subjects (Figure 3).

Further research focused on the association of gut dysbiosis in CAD patients with leakage of bacterial metabolites into the blood. The increasing trend in the number of *Gammaproteobacteria* in the CAD group has suggested that endotoxemia may directly play a role in atherogenesis, either in the induction of inflammation negatively affecting the vascular endothelium. An increase in Gram-negative bacterial species may affect intestinal epithelium tight junctions leading to increased intestinal permeability and LPS translocation from gut to circulation [21]. Over 85 bacterial species express tryptophanase enzyme degrading dietary tryptophan to indole [22]. Most of them are included within *Firmicutes*, *Proteobacteria*, and *Actinobacteria* phyla [23]. These phyla showed an upward trend in patients with CAD in our study. Hence, we assumed that indole synthesis might be increased in the gut of the CAD patients.

**Figure 4:**
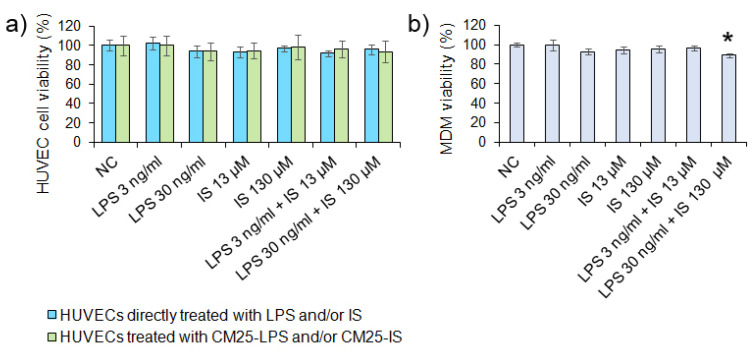
HUVECs and monocyte-derived macrophages (MDM) viability after stimulation with LPS and indoxyl sulfate (IS). (**a**) HUVECs and (**b**) MDM viability was assessed after 18 h with MTT. Data are means ± SEM of three independent experiments performed in triplicate. The asterisk above the bar indicates a statistically significant difference (*p* < 0.05).

**Figure 5:**
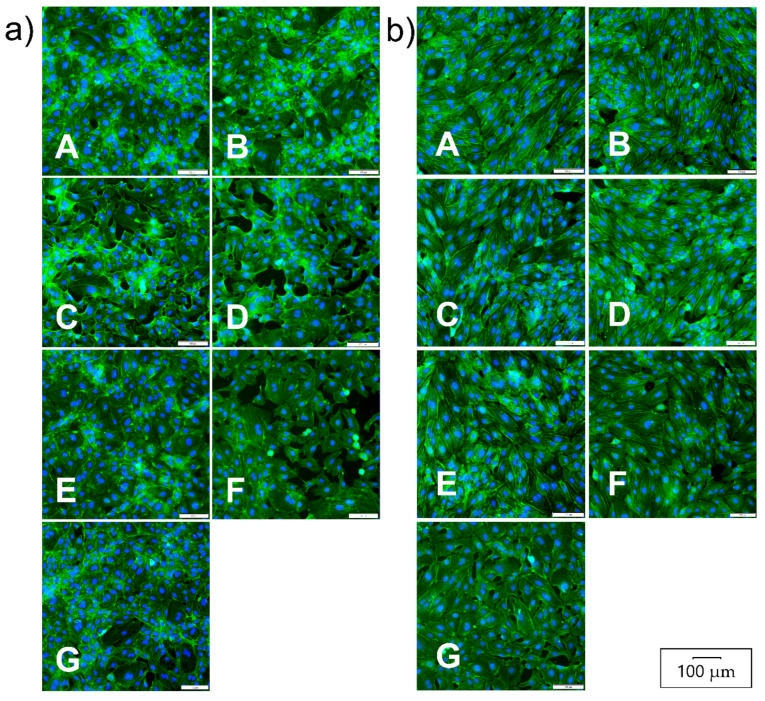
HUVECs morphology after stimulation with LPS and indoxyl. HUVECs were treated with LPS and IS or their combinations (**a**) or with conditioned medium from MDM (**b**) for 18 h. Fluorescence images present cells treated with LPS at 3 ng/mL and 30 ng/mL (**Panels A**,**B**); cells treated with indoxyl at 13 µM and 130 µM (**Panels C**,**D**); cells treated with LPS 3 ng/mL + IS 13 µM and LPS30 ng/mL + IS 130 µM (**Panels E**,**F**); and untreated cells (**Panels G**). Cells were stained with phalloidin-FITC and DAPI to visualize the cell’s actin filaments and nuclei, respectively.

The mean indoxyl level in CAD patients sera (13.3 µM), although twice as high as in healthy subjects (6.8 µM), did not reach statistical significance (*p =* 0.41). The most likely reason was the small size of the population studied. In contrast, the mean LPS level in CAD patients’ sera (2800 pg/mL) was much higher than in sera from healthy subjects (*p <* 0.0001), confirming gut dysbiosis-induced endotoxemia in patients. The LPS levels in most control sera were below the LAL assay’s threshold, i.e., 10 pg/mL (Table 1). Further, the direct and indirect impact of LPS and indoxyl at concentrations detected in patients sera on the thrombogenicity of endothelial cells was examined. The direct effect of both metabolites on HUVECs was investigated by applying LPS and indoxyl to cultured HUVECs. The indirect effect was analyzed with CM25 from MDMs stimulated with appropriate concentrations of LPS and indoxyl. Neither CM25 medium nor LPS and indoxyl or their combinations affected HUVECs viability (Figure 4a). Both metabolites at concentrations studied did not affect MDM viability except the highest ones used in the study (Figure 4b). The LPS at 30 ng/mL combined with indoxyl at 130 µM decreased MDM viability by >10% (*p =* 0.04). The morphology of HUVECs treated with LPS was unchanged compared to untreated cells (Figure 5a). In turn, HUVECs treated with CM25 appeared more elongated with distinct actin filaments (Figure 5b).

**Figure 6:**
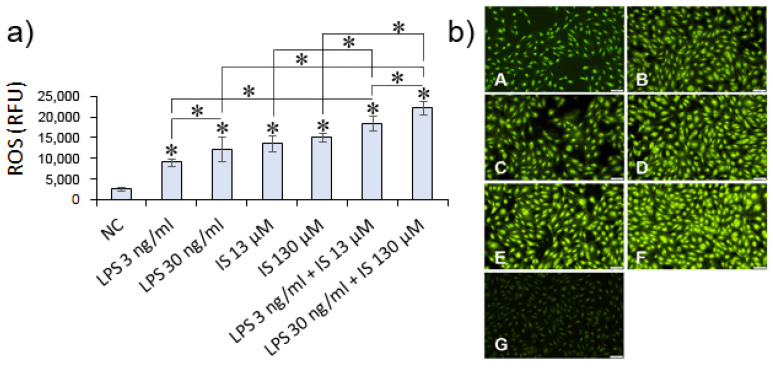
Reactive oxygen species (ROS) production. (**a**) HUVECs were treated for 5 h with LPS or IS or combined metabolites at appropriate concentrations. ROS was evaluated in HUVECs using a permeable probe H2DCF-DA compared to untreated cells (NC) as a negative control. Fluorescence intensity was read spectrophotometrically at Ex/EM = 488/525 nm. Data are presented as means ± SEM from three independent assays performed in triplicate. The asterisks above bars indicate statistically significant differences between negative control and metabolites examined. (**b**) ROS production was visualized in a fluorescent microscope. The image shows HUVECs treated with metabolites at the following concentrations: LPS 3 ng/mL and 30 ng/mL (**panels A**,**B**); IS 13 µM and 130 µM (**panels C**,**D**); LPS 3 ng/mL + IS 13 µM and LPS 30 ng/mL + IS 130 µM (**panels E**,**F**); and negative control (**panel G**).

Increased reactive oxygen species (ROS) production in endothelial cells is a hallmark of cardiovascular diseases and endothelial damage [24]. The study investigated whether low indoxyl and LPS concentrations present in patients sera could induce ROS in HUVECs. The results showed that LPS either at low and high concentrations (3 ng/mL and 30 ng/mL) increased ROS production in HUVECs of 160-fold (*p <* 0.00001) and >200-fold (*p <* 0.00001), respectively, compared to untreated cells. Indoxyl at both concentrations (13 µM and 130 µM) further enhanced ROS production induced by LPS, i.e., by >240-fold (*p <* 0.00001) and >260-fold (*p =* 0.00001), respectively. However, indoxyl alone at both concentrations 13 µM and IS 130 µM induced similar ROS levels in HUVECs (*p =* 0.25) (Figure 6).

Endothelial dysfunction induced by bacterial toxins and cytokines results in leukocyte-endothelial interactions and procoagulant activities, ultimately leading to thrombosis and atherosclerosis [25]. To determine whether LPS and indoxyl at concentrations detected in patients sera could affect ECs thrombogenicity, E-selectin and MCP-1 levels, and monocyte adherence to HUVECs was assessed. The results demonstrated that LPS at both concentrations induced similar low E-selectin levels in HUVECs although significantly higher (*p >* 0.05) than that determined in the negative control. On the contrary, IS induced higher E-selectin levels, i.e., 2.2-fold higher levels at a concentration 13 µM than LPS 3 ng/mL (*p =* 0.00001) and 1.7-fold higher levels at a concentration 130 µM than LPS 30 ng/mL (*p =* 0.00001). Both combined metabolites at low and 10-times higher concentrations (LPS 3 ng/mL + IS 13 µM and LPS 30 ng/mL + IS 130 µM) induced similar E-selectin levels (*p =* 0.08 and *p =* 0.22, respectively). These results suggested that indoxyl alone enhances E-selectin in HUVEC to a higher degree than LPS or its combination with IS.

**Figure 7:**
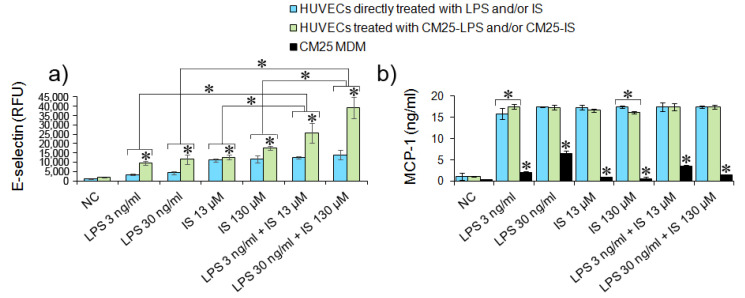
E-selectin and MCP-1 production in HUVECs stimulated with LPS and indoxyl. (**a**) E-selectin levels in HUVECs treated directly (blue bars) with LPS and IS or both metabolites and indirectly (green bars) with CM25-LPS/CM25-IS at appropriate concentrations. The asterisks above bars indicate the statistical differences in E-selectin levels between HUVECs stimulated directly with LPS/IS or indirectly with CM25. E-selectin was evaluated in HUVECs stimulated with LPS or IS or both bacterial metabolites for 18 h with FITC-conjugated mouse monoclonal anti-human E-selectin antibody. Data are presented as means ± SEM from two independent assays performed in quadruplicate. (**b**) MPC-1 levels in CM25 from MDM (black bars) and a culture media from HUVECs stimulated for 18 h with CM25 (green bars) or with LPS/IS (blue bars). The asterisks above black bars represent statistically significant differences between MCP-1 levels in CM25 and a culture media from HUVECs stimulated with LPS and IS for 18 h. The asterisks above white and grey bars indicate differences between MCP-1 levels in a culture media from HUVECs stimulated with CM25 and LPS/IS. MCP-1 was evaluated using the MCP-1 Human ELISA kit. Data are the means ± SEM from two independent assays performed in duplicate.

In contrast, CM25 exerted a much more significant effect on E-selectin levels in HUVECs, indicating that cytokines released by MDM stimulated with LPS and IS had a more pronounced impact on the endothelial expression of E-selectin (Figure 7a).

MCP-1 levels in CM25 used for HUVECs stimulation were significantly lower than in the culture media from HUVECs treated directly with LPS or indirectly stimulated with CM25 (*p <* 0.05) (Figure 7b). MDM treated for 18 h with indoxyl at 13 µM and 130 µM released significantly lower MCP-1 levels than cells treated with LPS (*p <* 0.05). Both metabolites combined at low concentrations (3 ng/mL LPS and 13 µM IS) induced higher MCP-1 levels than a combination of LPS and indoxyl at higher concentrations (30 ng/mL LPS and 130 µM IS) (*p <* 0.05). These results pointed out that indoxyl in a concentration-dependent manner inhibited MCP-1 secretion by MDM. Significantly higher MCP-1 levels in culture media from HUVECs directly stimulated with LPS and indoxyl than the levels in CM25 from MDM (*p <* 0.05) signify de novo production of the chemokine by HUVECs in response to bacterial metabolites. Furthermore, MCP-1 levels secreted by HUVECs were not affected by the concentration of LPS and indoxyl. At low and 10-times higher concentrations, both metabolites induced the secretion of comparable MCP-1 levels, ca. 16 ng/mL (*p >* 0.05), although significantly higher than that released by untreated cells (*p <* 0.05). These results indicated that endothelial cells produced and secreted MCP-1 upon stimulation with LPS and IS.

**Figure 8:**
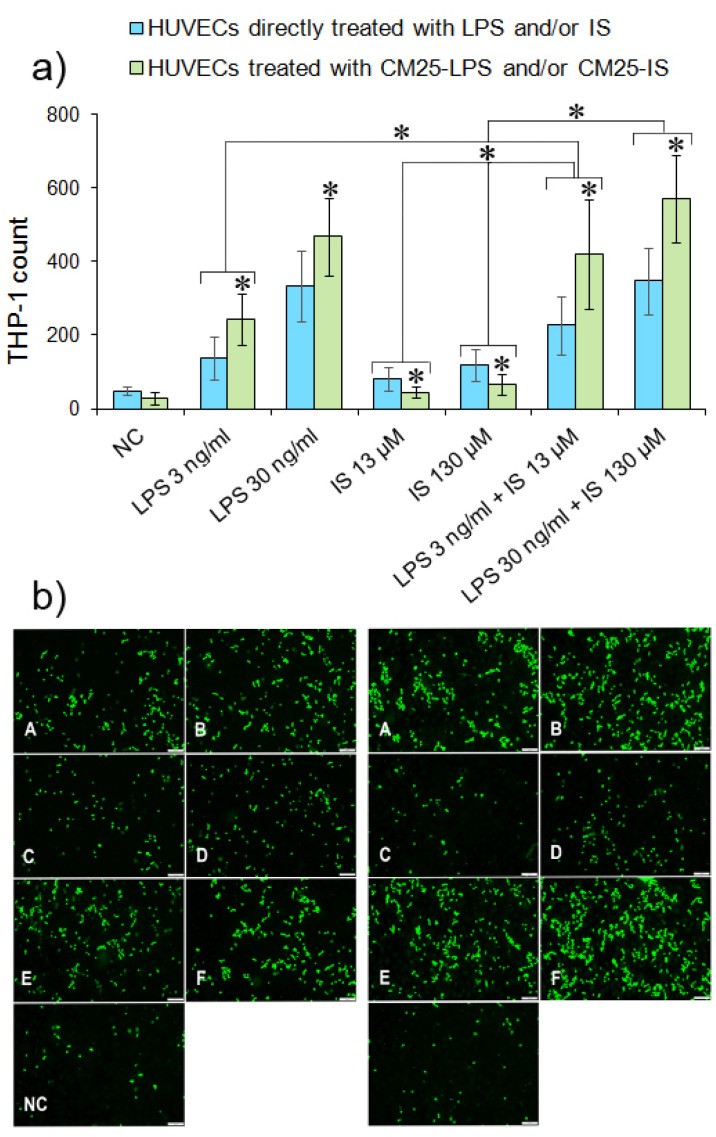
Monocyte adherence to HUVECs stimulated with LPS and indoxyl. (**a**) Quantitative THP-1 adherence to HUVECs treated directly (blue bars) with LPS/IS or indirectly (green bars) with CM25-LPS/CM25-IS at appropriate concentrations. The asterisks above bars indicate statistically significant differences between HUVECs treated directly and indirectly. The asterisks above white and grey bars indicate statistically significant differences between results for directly and indirectly treated cells. THP-1 cells stained with calcein-AM were incubated with HUVECs for 4 h and counted in a fluorescent microscope after washing out. (**b**) Fluorescence images of calcein-AM-stained monocytes adhering to HUVECs present HUVECs pretreated with LPS at 3 ng/mL and 30 ng/mL (**panels A**,**B**); cells treated with indoxyl at 13 µM and 130 µM (**Panels C**,**D**); cells treated with LPS 3 ng/mL + IS 13 µM and LPS30 ng/mL + IS 130 µM (**Panels E**,**F**); and untreated cells (NC).

Direct and indirect HUVECs treatment with LPS and indoxyl significantly augmented monocyte adhesion to endothelial cells compared to untreated cells (Figure 8). However, indoxyl at both concentrations reduced monocyte adherence to HUVECs compared to LPS at concentrations 3 ng/mL and 30 ng/mL (0.7-fold decrease for IS at 13 µM; *p =* 0.002 and 1.7-fold decrease for IS at 130 µM; *p =* 0.00001, respectively). Indoxyl at 13 µM combined with LPS 3 ng/mL increased monocyte adherence to HUVECs (*p =* 0.04), but when combined at 130 µM with LPS 30 ng/mL had no significant effect on THP-1 adherence comparing to LPS 30 ng/mL alone (*p =* 0.85). CM25 had a more pronounced effect on monocyte adhesion, indicating that the cytokines released by MDM stimulated with both metabolites enhanced monocyte adherence to endothelial cells. CM25-IS 13 µM reduced monocyte adherence to endothelial cells of 4.3-fold compared to LPS 3 ng/mL (*p =* 0.00001) whereas at 130 µM IS of 6.1-fold compared to LPS 30 ng/mL (*p =* 0.00001). Combination of low IS and LPS concentrations (LPS 3 ng/mL + IS 13 µM) increased monocyte adherence to HUVECs of 0.7-fold (*p =* 0.003) compared to LPS 3 ng/mL alone, but combination LPS 30 ng/mL + IS 130 µM had no impact on the adherence of THP-1 (*p =* 0.069) comparing to LPS at 30 ng/mL. These results suggested that IS, depending on the concentration when combined with LPS, enhances or reduces monocyte adherence to endothelial cells. Indoxyl combined with LPS at 3 ng/mL enhanced monocyte adherence, but combined with LPS at 30 ng/mL reduced adherence.

## 4. Discussion

An intact vascular endothelium displays an antithrombotic surface, preventing thrombosis and governing homeostasis. Endothelial dysfunction triggers a cascade of reactions shifting endothelial surface from antithrombotic to prothrombotic. As a result, endothelial activation promotes thrombus formation with two opposite implications for the host. First, the clot is digested and homeostasis restored; second, the thrombosis cascade progresses to CAD [26].

The gut microbiota plays a vital role in atherosclerosis, heart failure, diabetes, and obesity, acting as an independent risk factor [19]. A Western diet providing an excess of amino acids and proteins promotes the growth of proteolytic bacteria producing indole processed in the liver to indoxyl sulfate. Moreover, proteolytic gut microbiota metabolism favors gut dysbiosis [27]. Gut dysbiosis accompanies obesity. In the study, most CAD patients had a BMI close to 30, indicating obesity or at least prominent overweight. The gut microbiota of obese individuals contains a reduced proportion of *Bacteroidetes* and increased levels of *Firmicutes* [21], which was also confirmed in our study. Obesity leads to the dysregulation of the endocannabinoid tone system regulating gut permeability and plasma LPS levels. Muccini et al. [28] demonstrated that LPS acts as a master switch to control adipose tissue metabolism in vivo and ex vivo by blocking cannabinoid-driven adipogenesis. Obesity is associated with adipose tissue expansion and metabolic-associated fatty liver disease (MAFLD), characterized by a reduced ability of the liver to detoxify the blood from potentially toxic bacterial metabolites, i.e., LPS, leaking from the gut into the circulation [29]. This implies that obesity-associated gut dysbiosis leads to a cascade of reactions that promote atherosclerosis development and/or progression. 

The altered gut microbiota composition was confirmed in a metagenome study of patients with atherosclerosis and healthy individuals, strongly suggesting a correlation between CAD and dysbiosis [30,31,32].

Three enterotypes have been distinguished in gut microbiota based on key bacterial genera. The enterotypes are not related to age, weight, sex, or race but mostly depend on the long-term diet, allowing for better analysis of differences in the gut microbiome [19,20,33]. Enterotype I or *Bacteroides* enterotype is strongly associated with a Western diet rich in saturated fats and animal proteins. The Bacteroides enterotype comprises bacterial species that produce enzymes specialized in the degradation of animal proteins and characterizes increased saccharolytic and proteolytic capacity. Hence, the Bacteroides enterotype is more prevalent in industrialized countries.

In contrast, enterotype II or *Prevotella* enterotype is associated with bacteria producing hydrolases degrading plant fibers and of low lipophilic and proteolytic capacity. The *Prevotella* enterotype predominates in developing countries, in societies on agrarian diet [34,35]. The enterotype III or *Ruminococcus* enterotype, similarly to *the Bacteroides* enterotype, has been associated with protein- and fat-rich diet [36]. 

In the study *Prevotella* enterotype was under-represented, whereas *Ruminococcus* enterotype was over-represented in CAD patients compared to healthy subjects, supporting results of other studies [20,30,37,38]. 

The most striking difference in the gut microbiome composition was the significant depletion of *Bacteroidetes* in the CAD cohort, supporting previous observations [2,19,37]. *Bacteroidetes* play a pivotal role in maintaining a healthy gut ecosystem, stimulating immunity, providing short-chain fatty acids (SCFA), and facilitating the digestion of macronutrients [39]. *Bacteroidetes* depletion can result from a high-fat diet which changes the gut microbial profile and reduce its diversity. The depletion of *Bacteroidetes* combined with increased *Firmicutes* elevates circulating LPS levels leading to endotoxemia. Thus, *Bacteroidetes* may have the potential to regulate atherosclerosis progression [30]. Moreover, lipid A of LPS from *Bacteroides* is an antagonist of immune stimulation and inflammatory cytokine response [31,40]. Furthermore, the study demonstrated an increase in the prominent bacterial families producing indole, i.e., *Enterobacteriaceae*, *Clostridiaceae*, and *Verrucomicrobiace* in CAD patients, which although statistically insignificant, was correlated with an increased indoxyl level which was twice as high as in healthy subjects. 

Even though not reported in other studies, the prevalence of *Coriobacteriaceae* observed in the CAD cohort could play a role in the pathogenesis of atherosclerosis. In the animal model, the abundance of *Coriobacterium* correlated with higher cholesterol absorption from the gut [41]. At the genus level, in the CAD group, a marked decrease in *Alistipes* belonging to the family *Rikenellaceae* of *Bacteroidetes* was observed, supporting previous findings [41,42,43]. The *Alistipes* genus comprises species found primarily in the gut of healthy humans and is a potent SCFA producer essential for maintaining balanced gut microbiota [41]. According to various studies, *Alistipes* play a protective role in cardiovascular diseases [32,42,43].

However, the gut microbiome analysis in our study could be biased by the different abundance of both sexes in the CAD and HC groups. According to Kim et al. [44], sex is one of the critical variables affecting the gut microbiota. However, the hormone production declining with age seems to be less critical in the elderly. Moreover, in a well-designed study of a large Nederland cohort, sex explained only 0.5% of the total variation in the gut microbiota after correction of all confounding factors like diet, body mass index, and others [45]. Altogether, the results of studies concerning the differences in microbial taxa between the sexes are inconsistent. According to Ragonnaud et al. [46], some bacterial taxa, including *Bacteroides*, *Prevotella*, and *Ruminococcaceae*, do not correlate with sex, body mass index, and age. Hence, the diet seems to be a crucial factor shaping gut microbiota [44,45]. Considering that gut dysbiosis underlies the leakage of some bacterial metabolites into circulation, in the study, we analyzed levels of the endotoxin and indoxyl sulfate, a co-metabolite of indole in CAD patients sera, and the in vitro effects of these metabolites on vascular endothelium. The results showed that LPS and indoxyl combined at levels detected in CAD patients sera induced oxidative stress in cultured endothelial cells. Loffredo et al. [47] showed that LPS at a similar low concentration (3 ng/mL) induced oxidative stress in patients with peripheral arterial disease supporting our findings. Moreover, they found the correlation between LPS and zonulin of tight junctions, suggesting gut permeability as a trigger for LPS translocation. In turn, Lee et al. [48] demonstrated that indoxyl at low concentrations (50 µg/mL) induced significant ROS production in HUVECs. On the other hand, Dou et al. [49] demonstrated that indoxyl at concentrations from 25 µg/mL to 50 µg/mL increased ROS production in HUVECs only insignificantly, which contradicts our results. However, these discrepancies may have been due to methodological variations, e.g., different endothelial cell lines or different culture media.

Oxidative stress is a potent risk factor for atherogenesis. Pathological endothelial activation results in the secretion of factors attracting monocytes and induces the expression of monocyte-binding adhesion molecules [50]. E-selectin plays an essential role in priming and amplifying the innate immune response of endothelial cells, leukocyte rolling, and adhesion [51]. LPS directly increases the expression of E-selectin and integrin counterpart receptors in ECs [6]. In the study, LPS and indoxyl applied directly and indirectly in conditioned medium to endothelial cells increased E-selectin levels reaffirming earlier findings [52]. Higher E-selectin levels induced by CM25 in HUVECs indicated that cytokines and other factors released by MDM upon treatment with LPS and IS enhance the effects of these metabolites on E-selectin expression by endothelial cells. These results implied that inflammation increases endothelial thrombogenicity induced by bacterial metabolites leaking from the gut.

MCP-1 is a significant chemokine recruiting monocyte/macrophage to the injured vascular endothelial cells. LPS activates endothelial cells and monocyte/macrophage to secrete proinflammatory cytokines, i.e., IL-6 and IL-8, and MCP-1 among them [53,54]. In the study, LPS and indoxyl sulfate combined induced synthesis de novo and secretion of MCP-1 in HUVECs. On the contrary, indoxyl alone in a concentration-dependent manner inhibited MCP-1 secretion by macrophages but not by endothelial cells, which corroborates results from other studies. Ferreira et al. [55] demonstrated that indoxyl at a 53 µg/mL concentration decreased MCP-1 production by monocytes. In contrast, according to Tumur et al. [56], indoxyl increased MCP-1 expression in HUVECs but only at a concentration ≥500 µM. Despite methodological differences in the studies, these contradictory results point out the necessity for further study of the impact of indoxyl on MCP-1 production in endothelial cells.

Considering the deleterious effect of LPS and indoxyl on ROS production, MCP-1, and E-selectin levels in endothelial cells demonstrated in the study, the infuence of both metabolites on monocyte adherence was further examined. Four hours of exposure of endothelial cells to LPS or CM25 significantly increased monocyte adhesion. However, this effect diminished when HUVECs were stimulated with indoxyl or the combination of LPS and indoxyl at higher concentrations. According to Ito et al. [57], indoxyl at concentrations below 25 µM generated insignificant adherence of THP-1 to HUVECs. However, the adherence of monocytes increased in the presence of tumor necrosis factor-α (TNF-α), which agrees with our results. In the study, conditioned medium containing cytokines released by MDM stimulated with LPS and indoxyl enhanced monocyte adhesion to HUVECS compared to these metabolites directly applied to endothelial cells. Altogether, these data suggest a cytokine- and exposure-time-dependent effect of indoxyl on monocyte adhesion to vascular endothelium.

It has long been known that LPS and indoxyl induce inflammation [58,59]. However, findings to date refer to high or even very high LPS and indoxyl concentrations accompanying sepsis or CKD, which rarely relate to the actual blood levels of both metabolites in CAD patients without kidney failure. Similarly, studies of indoxyl toxicity to endothelium are based on high concentrations of the metabolite detected in CKD patients sera. For the first time, the study attempted to evaluate the effects of a combination of low concentrations of endotoxin and indoxyl on vascular endothelium. The results obtained strongly suggest that both these metabolites, even in meager concentrations, exert a deleterious effect on endothelial cells’ thrombogenicity. A significant limitation of the study is the small study cohort associated with strict recruitment criteria excluding patients with various chronic diseases often associated with CAD.

Nonetheless, the study indicated that bacterial metabolites leaking from the gut adversely affect vascular endothelium, promoting the development or progression of CAD. Moreover, the study demonstrated the co-toxicity of bacterial metabolites entering blood during gut dysbiosis and the impact of cytokines released by monocytes upon stimulation with endotoxin and indoxyl on the enhanced detrimental effect of these metabolites on the endothelium. Although the study hardly reflects the scenario in vivo, the results provide new insights into the role of gut dysbiosis in atherogenesis and the necessity for further study of the co-toxicity of bacterial metabolites, primarily upon inflammation.

## 5. Conclusions

16S rRNA sequencing analysis revealed gut dysbiosis in CAD patients that was further confirmed by elevated levels of bacterial metabolites, i.e., LPS and indoxyl sulfate in patients sera. Both metabolites demonstrated co-toxicity in meager concentrations to endothelial cells inducing ROS, E-selectin, and MCP-1 production and promoting thrombogenicity of endothelial cells confirmed by monocyte adherence.

## Figures and Tables

**Table 1:** Baseline clinical characteristics of the study cohort.

	CAD (Average) *N* = 15	Range	HC (Average) *N* = 15	Range	*p*-Value
Age (years)	67.2 ± 9	54–90	57 ± 11.1	47–74	*p >* 0.5
Sex(male/female)	11 4		5 /10		
BMI	29.4 ± 5.05	23.6–39.26	25.95 ± 4.21	18.71–36.25	*p <* 0.05
CAD type	10 SA; 5-ACS		NO		
Indoxyl sulfate (µM)	13.1 ± 4.1	3.6–21.2	6.9 ± N3.5	2.1–15.7	*p =* 0.41
LPS (pg/mL)	2800 ± 1500	900–5500	8.5 ± 12.3	0.0–50	*p <* 0.0001

CAD, coronary artery disease; HC, healthy controls, SA—stable angina, ACS—acute coronary syndrome.

## Data Availability

Data supporting results will be made available by the authors upon request when warranted.
